# Rethinking headache as a global public health case model for reaching the SDG 3 HEALTH by 2030

**DOI:** 10.1186/s10194-023-01666-2

**Published:** 2023-10-27

**Authors:** Paolo Martelletti, Matilde Leonardi, Messoud Ashina, Rami Burstein, Soo-Jin Cho, Augustina Charway-Felli, David W. Dodick, Raquel Gil-Gouveia, Licia Grazzi, Christian Lampl, Antoinette MaassenVanDenBrink, Mia T. Minen, Dimos Dimitrios Mitsikostas, Jes Olesen, Mayowa Ojo Owolabi, Uwe Reuter, Elena Ruiz de la Torre, Simona Sacco, Todd J Schwedt, Gianluca Serafini, Nirmal Surya, Cristina Tassorelli, Shuu-Jiun Wang, Yonggang Wang, Tissa Wijeratne, Alberto Raggi

**Affiliations:** 1 Department of Clinical and Molecular Medicine, Sapienza University, Rome, Italy; 2 Neurology, Public Health and Disability Unit, Fondazione IRCCS Istituto Neurologico Carlo Besta, Via Celoria 11, Milan, 20133 Italy; 3 Department of Neurology, Danish Headache Center, Rigshospitalet Glostrup, Faculty of Health and Medical Sciences, University of Copenhagen, Copenhagen, Denmark; 4 John Hedley-Whyte Professor of Anesthesia and Neuroscience at the Beth Israel Deaconess Medical Center and Harvard Medical School, Boston, MA USA; 5 Department of Neurology, Dongtan Sacred Heart Hospital, Hallym University College of Medicine, Military Hospital, Hwaseong, Korea; 6 37 Military Hospital, Accra, Ghana; 7 Department of Neurology, Mayo Clinic, Scottsdale, AZ USA; 8 Neurology Department, Hospital da Luz Headache Center, Hospital da Luz Lisboa., Lisbon, Portugal; 9 Center for Interdisciplinary Research in Health, Universidade Católica Portuguesa, Lisbon, Portugal; 10 Neuroalgology Unit and Headache Center, Fondazione IRCCS Istituto Neurologico Carlo Besta, Milan, Italy; 11 Department of Neurology and Stroke Unit, Koventhospital Barmherzige Brüder Linz, Linz, Austria; 12 Headache Medical Center Linz, Linz, Austria; 13 Division of Vascular Medicine and Pharmacology, Department of Internal Medicine, Erasmus MC University Medical Center, Rotterdam, Netherlands; 14 Department of Neurology, NYU Langone Health, NY New York, USA; 15 1st Neurology Department, Eginition Hospital, Medical School, National and Kapodistrian University of Athens, Athens, Greece; 16 Faculty of Clinical Sciences; Center for Genomic and Precision Medicine, College of Medicine,, University of Ibadan, Ibadan, Nigeria; 17 Department of Neurology, Charité Universitätsmedizin Berlin, Berlin, Germany; 18 Universitätsmedizin Greifswald, Greifswald, Germany; 19 European Migraine and Headache Alliance, Brussels, Belgium; 20 Department of Biotechnological and Applied Clinical Sciences, University of L‘Aquila, L‘Aquila, Italy; 21 Department of Neuroscience, Rehabilitation, Ophthalmology, Genetics, Maternal and Child Health (DINOGMI), Section of Psychiatry, University of Genoa, Genoa, Italy; 22 IRCCS Ospedale Policlinico San Martino, Genoa, Italy; 23 Surya Neuro Centre Mumbai, Mumbai, India; 24 Department of Brain and Behavioral Sciences, University of Pavia, Pavia, Italy; 25 Headache Science and Neurorehabilitation Center, IRCCS Mondino Foundation, Pavia, Italy; 26 Brain Research Center, National Yang Ming Chiao Tung University, Taipei, Taiwan; 27 Department of Neurology, The Neurological Institute, Taipei Veterans General Hospital, Taipei, Taiwan; 28 Headache Center, Department of Neurology, Beijing Tiantan Hospital, Capital Medical University, Beijing, China; 29 Department of Neurology, Sunshine Hospital, St Albans, VIC Australia

**Keywords:** Migraine, Medication overuse headache, Tension-type headache, Sustainable development goals, Global burden of disease study, Low- and middle-income countries

## Abstract

The 2030 Agenda for Sustainable Development sets out, through 17 Sustainable Development Goals (SDGs), a path for the prosperity of people and the planet. SDG 3 in particular aims to ensure healthy lives and promote well-being for all at all ages and includes several targets to enhance health. This review presents a “headache-tailored” perspective on how to achieve SDG 3 by focusing on six specific actions: targeting chronic headaches; reducing the overuse of acute pain-relieving medications; promoting the education of healthcare professionals; granting access to medication in low- and middle-income countries (LMIC); implementing training and educational opportunities for healthcare professionals in low and middle income countries; building a global alliance against headache disorders. Addressing the burden of headache disorders directly impacts on populations’ health, as well as on the possibility to improve the productivity of people aged below 50, women in particular. Our analysis pointed out several elements, and included: moving forward from frequency-based parameters to define headache severity; recognizing and managing comorbid diseases and risk factors; implementing a disease management multi-modal management model that incorporates pharmacological and non-pharmacological treatments; early recognizing and managing the overuse of acute pain-relieving medications; promoting undergraduate, postgraduate, and continuing medical education of healthcare professionals with specific training on headache; and promoting a culture that favors the recognition of headaches as diseases with a neurobiological basis, where this is not yet recognized. Making headache care more sustainable is an achievable objective, which will require multi-stakeholder collaborations across all sectors of society, both health-related and not health-related. Robust investments will be needed; however, considering the high prevalence of headache disorders and the associated disability, these investments will surely improve multiple health outcomes and lift development and well-being globally.

## Introduction

In 2015 all United Nations Member States adopted the 2030 Agenda for Sustainable Development (ASD-2030) which sets out, through 17 Sustainable Development Goals (SDGs), a path for the prosperity for people and the planet [1]. Specifically, the SDG 3 is aimed to “Ensure healthy lives and promote well-being for all at all ages” and is articulated in a set of targets which are overall aimed to reduce mortality and disease burden.

To pursue the goal of SDG 3, headache disorders should be adequately addressed at a global level, as they are among the most prevalent and disabling conditions: primary headaches constitute approximately 90% of headache cases, and the remaining are secondary. According to the latest estimates of the Global Burden of Disease Study (GBD), in 2019 there were 793.8 million incident cases of primary headaches, 2.60 billion prevalent cases and a total of 46.6 million Years Lived with a Disability (YLD) [2]. In terms of YLD rates, globally, headache disorders rank 3rd after low back pain and depressive disorders; however, amongst persons aged 15–49 they rank 1st, and account for 8% of total YLDs. However, primary headaches are also frequent and disabling in children and adolescents [3]. Globally, and considering all-age populations, tension-type headache (TTH) is the second most prevalent condition, and migraine the second most disabling [2]. Despite the fact that headaches are not associated to fatal outcomes, if these are taken into account (and thus Disability-Adjusted Life Years – DALYs rankings are considered) headache disorders burden is still considerable. In fact, they rank 15th considering all-age group, 2nd considering the population aged 10–24 years (where they account for 5% of the total DALYs) and 5th considering the population aged 25–49 years (where they account for 3.7% of the total DALYs) [2].

Headache disorders are long-lasting conditions, which usually peak in the first adulthood particularly among women [4] thus magnifying gender inequalities. Migraine in particular poses a relevant burden on populations due to its considerable prevalence (around 14%) and substantial impact, with symptoms peaking during the most productive years. TTH, although less disabling, is very frequent, to the point that the majority of the population experiences it in their lifetime [5]. In addition to this, secondary headaches, and particularly those associated with long-COVID syndrome, might further increase the overall prevalence of these non-communicable diseases [6–9].

Reducing the burden of headaches is a way to ensure healthier lives to approximately one third of the world population. However, considering the heterogeneity in presentation and the variability in frequency, a multiplicity of parameters has to be taken into consideration to ease the overall burden of headache disorders [10].

The aim of this narrative review is to propose a set of actions that can be implemented in order to reduce the burden and disability of headache globally, by proposing a way to rethink how to scale and implement actions using headache as a public health target towards SDG 3 by 2030. The actions herein discussed are not to be intended as practical solutions, but as proposals to set the stage on policy guidelines. The review is organized into six subsections, each addressing the topic of a specific SDG 3 target (see Table 1 for a synopsis):

**Table 1:** A Synopsis on SDG 3 targets implementation reducing the burden of headache disorders with a synthesis of main policy actions

SDG3 Target	Proposal of implementation for reducing the burden headache disorders	Main policy actions
**Target 3.4**: By 2030, reduce by one third premature mortality from non-communicable diseases through prevention and treatment and promote mental health and well-being	Reducing the burden of primary headaches by reducing chronification, reducing barriers and impact on daily life in a biopsychosocial perspective	1. Developing a pathway of primary headaches healthcare to treat patients according to their specific clinical severity and needs. 2. Defining clinical severity not only with headache frequency. 3. Recognizing lifestyle issues and habits, as well as demographic issues, as elements of interest.
**Target 3.5**: Strengthen the prevention and treatment of substance abuse, including narcotic drug abuse and harmful use of alcohol	Reducing medication overuse in acute management of primary headaches: strategies at primary, secondary, tertiary levels of care in a global perspective	1. Preventing MO through identification of risk factors. 2. Reducing the use of drugs, especially opioids, that might increase the risk of MO. 3. Promoting neurology training in LMIC. 4. Addressing social and cultural factors that determine low access to headache care, including stigma.
**Target 3.8**: Achieve universal health coverage, including financial risk protection, access to quality essential health-care services and access to safe, effective, quality and affordable essential medicines and vaccines for all	Promoting education of health care professionals in the management of primary headaches and defining feasible methodology to support health-care facilities development to deliver comprehensive headache care pathways	1. Plan and implement specific headache training directed to either medical doctors and pharmacists in LMIC, which incorporates: 1.a. Few issues on pathogenesis; 1.b. Extensive information on clinical aspects and differential diagnoses; 1.c. Detailed account of the available pharmacological and non-pharmacological treatments that are available and affordable in the single contexts, and lifestyle issues.
**Target 3.b**: Support the research and development of vaccines and medicines for the communicable and non-communicable diseases that primarily affect developing countries, provide access to affordable essential medicines and vaccines, in accordance with the Doha Declaration on the TRIPS Agreement and Public Health, which affirms the right of developing countries to use to the full the provisions in the Agreement on Trade-Related Aspects of Intellectual Property Rights regarding flexibilities to protect public health, and, in particular, provide access to medicines for all	Defining strategies for access to existing treatments for headaches in low- and middle-income countries and for facilitating the inclusion of these countries in the research and development of new medicines (RCTs or RWS)	1. Improving care and access to preventive treatment through: public education; improved training of primary care physicians; improved training in the residency program for neurology; increased reach of neurologists to the rural areas. 2. Fostering research, particularly through RWS, to identify the performance of different treatment in specific contexts and enable define context-specific recommendations.
**Target 3.c**: Substantially increase health financing and the recruitment, development, training and retention of the health workforce in developing countries, especially in least developed countries and small island developing States	Defining strategies to develop and implement training and education in low- and middle-income countries to improve the skills of healthcare professionals for management of headaches in primary and secondary care	1. Implement the local headache school by means of a short course formats, which include the training on the use of primary headache clinical diagnostic criteria. 2. Implement or create the local headache group by means of on-site (hospital) training and mentoring. 3. Integrate the “training of the trainers” methodology by collaborating with local academic institutions. 4. Secure support from the Ministries of Health to ensure participation in the training. 5. Train healthcare workers living in the rural setting, to improve recruitment and retention of skilled healthcare providers in rural underserved areas.
**Target 3.d**: Strengthen the capacity of all countries, in particular developing countries, for early warning, risk reduction and management of national and global health risks	Defining strategies for an inclusive and global alliance against headache disorders among headache healthcare professional working parties to respond to public health unmet needs in headache area	1. Enhance collaboration between scientific and political stakeholders in order to reach the following: 1.a. Increasing research capacity, by facilitating access to funding for those working in LMIC and limiting the price of open-access publications; 1.b. Promoting e-education to increase inclusiveness of all possible healthcare workers; 1.c. Promoting access to meeting and congresses of gender-, age-, and ethnicity-balanced representatives, and promoting patient’s representatives’ participation; 1.d. Increasing the use of telemedicine 1.e. Studying common solutions to allow innovative drugs to be available also in LMICs.

*MO *Medication Overuse, *LMIC *Low- and Medium-Income Countries, *RCT *Randomized Controlled Trials, *RWS *Real World Studies

1. Reducing the burden of primary headaches by reducing chronification, reducing barriers and impact on daily life in a biopsychosocial perspective (Target 3.4).
2. Reducing medication overuse in acute management of primary headaches: strategies at primary, secondary, tertiary levels of care in a global perspective (Target 3.5).
3. Promoting education of health care professionals in the management of primary headaches and defining feasible methodology to support health-care facilities development to deliver comprehensive headache care pathways (Target 3.8).
4. Defining strategies for access to existing treatments for headaches in low- and middle-income countries and for facilitating the inclusion of these countries in the research and development of new medicines (RCTs or RWS) (Target 3.b).
5. Defining strategies to develop and implement training and education in low- and middle-income countries to improve the skills of healthcare professionals for management of headaches in primary and secondary care (Target 3.c).
6. Defining strategies for an inclusive and global alliance against headache disorders among headache healthcare professional working parties to respond to public health unmet needs in headache area (Target 3.d).

What we propose here does take into account any “standard” state of headache care as a starting point for the implementation of policies first and actions then. In fact, the inequalities at the global level are so wide that in the most disadvantaged countries, i.e. low- and middle-income countries (LMIC), the possibility of seeing a healthcare professional with specific expertise on headaches is very low, and headache care is mostly based on anti-inflammatories. Setting the stage for policies, particularly in LMIC, is a priority that clearly comes before concrete actions can be even planned, but it is of outmost importance considering that around 80% with headache disorders are from LMIC [2].

### Reducing the burden of primary headaches by reducing chronification, reducing barriers and impact on daily life in a biopsychosocial perspective (Target 3.4)

Primary headache disorders are common and burdensome conditions. Considering all-age groups, TTH is the second most prevalent condition, and migraine the second most disabling. In the age group 15–49, headache disorders rank first accounting for approximately 8% of the total disability [2].

Chronification is the process which leads to an increase in headache frequency above 15 days per month and is associated with more severe disability [11]. The main predictors of chronification, with specific reference to migraine, are: comorbidities, genetic predisposition, psychological and lifestyle factors, and medication overuse [11, 12]. When compared to those with episodic migraine (EM), patients with chronic migraine (CM), high-frequency episodic migraine, and chronic TTH show higher disability and impact, as shown in different recent studies [13–19], lower treatment satisfaction, and higher treatment needs which are often not adequately met in the clinical practice [17–19].

When patients are seen in the healthcare system at a single point in time, measuring headache frequency might not adequately assess the severity of the patient’s condition [16]. In fact, excluding those with lower frequency (e.g. up to 4 days per month) or those with the highest frequency (i.e. daily or close to daily), a snapshot of severity solely based on frequency is only partially informative, as it does not address the impact of the attacks nor whether the patient’s clinical situation is improving or worsening. For example, a frequency of 15 headache days per month might reflect substantial improvement, if the baseline was 25–30 days, or reason for alarm if the baseline was 4–8 days, and fluctuations between EM and CM are in particular very common. As shown by Serrano and colleagues, 7.6% of patients with EM progress to CM, and nearly 75% of those with CM may remit to EM at some point during a 12-month period [20].

A full evaluation of clinical severity, which in turn is relevant to inform how to reduce chronification by addressing barriers and impact on daily life, needs to be based on a biopsychosocial perspective [21] and take into account a multiplicity of parameters [16]. These should at least include longitudinal changes in headache frequency, current headache frequency, headache severity, associated symptoms (e.g. nausea, osmophobia, phono- and photophobia cutaneous allodynia), the presence of aura, TTH-like pain, comorbidities, psychological difficulties, the variability in response to treatment, and the degree to which pain and other symptoms limit the ability of patients to function at their usual level, which can be fruitfully measured using validated instruments addressing headache-related disability, quality of life instruments, or headache impact. Lack of access to appropriate healthcare is a major barrier to good patient outcomes, including lack of access to medical consultation, accurate diagnosis, and the most appropriate pharmacological and non-pharmacological treatments [22]. Lack of access also prevents appropriate diagnosis and management of common comorbidities, including but not limited to psychological comorbidities that are common amongst those with chronic headache and can increase the complexity of the treatment approach [23–25].

Unmet medical needs are commonly reported by patients with headache disorders, especially migraine, and are often focused on the lack of adequate therapies [18]: this, however, does not strictly deal with the availability of “a tailored therapy for a patient”. In fact, the armamentarium of medications for acute and preventive headache care is huge, although its availability varies by country, and may be complemented by non-pharmacological treatments. Therefore, the challenge deals with the identification of the best care pathway for each patient, also considering the setting in which they are treated, i.e. treating them at the “appropriate level of care”. The majority of patients with primary headaches may be treated at a primary (nurse or doctor-based, according to setting) or intermediate level of care [26], leaving specialty care for the most complex cases only: the implementation of a model which is based on both clinical severity, patients’ needs, and response to available treatments is expected to be cost-effective and cost-saving in the medium-term [27].

In summary, barriers mostly deal with the organization of healthcare systems, which might hinder the ability to provide the best treatment, but also with “cultural” issues, i.e. physicians often lack training in how to best treat patients. In the latest years, numerous different treatments and approaches have demonstrated their efficacy as preventive treatments for patients with migraine disorders, including pharmacological [28] and non-pharmacological strategies [29]. The current challenge, therefore, deals with delivering the most appropriate evidence-based treatment at the most appropriate level of care for each single patient.

The main policy action to be taken is therefore to develop, at the level of local health systems, a pathway of primary headaches healthcare which is able to identify patients according to their specific clinical severity and needs, e.g. patients with chronic headache with or without medication overuse for whom headache frequency reduction and cessation of medication overuse is the target, as opposed to those with a stable pattern of low-frequency episodic migraine, for whom maintenance of such pattern and avoiding chronification is the target. In the case of migraine, such actions should include:

1. A definition of clinical severity which accounts not only for frequency-based parameters, but also for the associated migraine symptoms – such as nausea, osmophobia, phonophobia, photophobia, cutaneous allodynia, aura – the quality and intensity of pain, the presence of comorbidities, and the degree to which symptoms’ severity limit patients’ ability to function at their usual level in their daily lives [10];
2. The appreciation and consideration of recent (i.e. referred to the last 6–12 months) variations in the parameter “headache frequency” which accounts for the increasing and decreasing trend. This should not only be valid in clinical settings but should also be implemented in research (e.g. among inclusion criteria for RCTs) [10];
3. A guideline for the recognition of patients’ needs which goes beyond the simple “get rid of headache” approach to embrace a biopsychosocial perspective, which fully accounts not only for patients’ clinical features, but also for socio‑demographic and lifestyle factors, including socioeconomic status, working environment, tasks and habits, lifestyle issues such as diet, sleep pattern, engagement in exercise, and presence of external stressors [14].

Achieving such a comprehensive migraine healthcare pathway is of primary relevance, as it may not only improve patients’ health, reduce their disability and enhance their quality of life, but it is expected to produce a significant reduction of disease costs [27, 30, 31].

### Reducing medication overuse in acute management of primary headaches: strategies at primary, secondary, tertiary levels of care in a global perspective (Target 3.5)

One of the major goals of the SDG 3 by 2030 campaign is the treatment of substance abuse. Patients with chronic headache (≥ 15 headache days per month for > 3 months) frequently overuse symptomatic medications, a form of excessive intake of drugs which might drive to the development and maintenance of medication overuse headache (MOH) [31]. MOH affects 1% of the global population and is listed as a secondary headache disorder in the International Classification of Headache Disorders, 3rd edition (ICHD-3) [4]. MOH is best defined as the sequela of an inadequately managed aggressive type of primary headache, coupled with the increased use of symptomatic medications, lifestyle factors and genetic predisposition [32, 33]. Medication overuse is defined as the use of symptomatic medications for the treatment of headache on ≥ 15 days or ≥ 10 days per month, depending on the class of overused medication [4]. Commonly overused symptomatic medications for the treatment of headache include nonsteroidal anti-inflammatory drugs (NSAIDs), triptans, ergot alkaloids, barbiturates, and opioids [34]. Additionally, MOH is associated with high levels of disability, high healthcare spending, and increased healthcare consumption [27]. To reduce the burden of MOH globally, strategies targeting the primary, secondary, and tertiary level of care must be implemented. Strategies that are necessary to reach the goals of the SDG 3 by 2030 campaign include increasing primary care education in the diagnosis and treatment of common headache disorders, expanding the development of adequate care delivery systems for the treatment of headache in developing nations, and reducing the social stigma of headache and substance abuse. In addition to this, making available medications other than NSAIDs and paracetamol in those countries where other drugs are not available or affordable is also needed.

It cannot be ignored that the development of chronic and complicated headache associated to medication overuse is due to several factors, including comorbidities, genetic predisposition, psychological and lifestyle factors, and type of acute medication used [11, 12]. Among lifestyle issues, adequate sleep, eating, hydration and physical activity are the ones that can be easily tackled at all levels of care: in most cases, these are next to zero cost interventions, that are therefore particularly suitable for LMIC. Although the pathogenesis of MOH is poorly understood, most cases of MOH are associated with a progressive clinical course from EM to CM, in turn associated to the excessive consumption of medications [35]. This is supported by findings that medication overuse is present in 30% to more than 50% of patients with CM, defined as ≥ 15 headache days per month, for > 3 months, in which ≥ 8 headaches demonstrate characteristics of migraine [4, 36–40]. Early recognition and prescription of appropriate abortive and preventative treatment of EM at the primary care level is crucial to reduce the risk of MOH. Additionally, the treatment of MOH is complex and involves withdrawal from the overused medication [35, 41]. The concomitant use of preventative medications during detoxification may be useful in the treatment of MOH evolved from EM, but further research is needed to determine the efficacy of this approach [41–44].

Headaches are common in primary care, representing 1.5% of cases seen by general practitioners (GP) [45]. Multiple studies have demonstrated GPs’ discomfort with both the diagnosis and treatment of various primary headache disorders [46, 47]. Furthermore, recent studies demonstrated a significant underutilization of preventative medication, reporting that only 16.8% of the eligible 40.4% of migraine patients use preventative medication in the US [48] and in population setting in Europe, less than 15% of 33.8% eligible patients were treated with preventives by their GPs [49]. Underutilization of preventive medications is associated with a compensatory use of symptomatic medications at higher frequencies, increasing the potential for substance overuse and MOH [34]. To reduce the incidence of MOH, it is crucial that patients that are eligible for preventive therapy are recognized and treated early throughout the clinical course. To achieve this goal, increased education campaigns targeting patients and healthcare workers and providers of all levels should be implemented with clear guidelines describing patients that are eligible for preventive migraine therapy. This should be accompanied by policy actions to make preventive treatments which demonstrated an acceptable control over migraine activity available and affordable in all countries.

An additional concern at the primary care level is the inappropriate prescription of medications such as barbiturates, ergot alkaloids, and opioids for the acute treatment of migraine. Although many physicians continue to prescribe these medications, their use should be restricted since substantial research has demonstrated an increased risk of clinical progression and a high risk of MOH associated with these medications [50]. Rates of opioid abuse and mortality continue to rise within the USA, a phenomenon referred to as the opioid epidemic, claiming an estimated 100,000 lives per year [51]. Notwithstanding the poor efficacy of opioids in migraine and the high rates of progression to MOH associated to their use, recent research reported that 36.3% of individuals enrolled in a US population study used opioids for the symptomatic treatment of migraine [52]. This is a critical concern that requires vigorous physician education and prescribing restrictions to reduce the burden of opioid exposure for those with migraine. Additionally, patient education should involve discussion regarding the need to limit the use of symptomatic migraine medications such as NSAIDs and triptans, to a maximum number of days/month and the risks associated to medication overuse.

There are multiple barriers related to the optimal treatment of patients with MOH in developing nations, one of the leading being access to physicians trained in the diagnosis and treatment of headache, due to a lack of care delivery systems at all levels of headache treatment [46], and lack of access to appropriate medication. Neurologists are the physicians that are most likely to gain specific training in the diagnosis and treatment of headache, yet many developing nations lack the financial or institutional capabilities to support specialized medical training. Strikingly, developing nations in South-East Asia and Africa report 0.04 to 0.1 neurologists per 100,000 citizens, while in Europe the ratio is 6 per 100,000 [53]. Additionally, due to a lack of tertiary level care, scarce research has been completed regarding the epidemiology and burden of headache within developing nations [54]. Without high quality data guiding specific interventions of headache management within these nations, inadequate care is bound to occur, thus increasing the risk of MOH. To reduce the burden of MOH in these developing nations, additional neurology training programs should be economically and institutionally supported with the goal of designing an efficient headache care delivery system. The possibility to implement a simple enough training allowing those healthcare officers with education up to bachelor level to diagnose and treat the most common headache by asking the simplest question possible should also be explored.

In both developed and developing nations, headache is an underreported condition due to multiple social and cultural factors [47]. Beliefs that headaches are predominantly associated with psychiatric pathology, emotional dysregulation, visual impairments, cardiovascular disease, and various infectious diseases decreases the likelihood that patients will seek medical care for primary headaches [47]. These social factors act as a barrier to the prevention of MOH. Nevertheless, headache is still a leading complaint in neurology clinics, ranging from 7.7 to 31.9% of all clinic visits in African countries and from 4 to 29.3% in Asian and South-Asian ones [55–64].

Another concern is the stigma attributed to illicit substance abuse, relevant to both developed and developing nations [65], which is an underreported and undertreated disorder associated with high mortality [66]: acute medication overuse for headaches is not listed among the causes of mortality, however, the overuse of opioids for headaches might claim victims that end up lost in the cauldron of substance abuse associated with high mortality. Although substance overuse associated with MOH is predominantly linked to the use of prescription and over-the-counter medications, high social pressures, and stigmatization against the adequate treatment for substance abuse is detrimental to patients. To reduce the burden of MOH within the global population, public education campaigns regarding the early recognition and treatment of substance overuse are necessary.

### Promoting education of health care professionals in the management of primary headaches and defining feasible methodology to support health-care facilities development to deliver comprehensive headache care pathways (Target 3.8)

The development of a framework to educate healthcare professionals on headache and to support the development of health care facilities for taking care of people who have frequent headaches is extremely important. In this regard, important information is provided by available surveys developed within the Global Campaign against Headache project [26, 27, 67].

This program showed that in most countries of Europe there are headache centers with highly specialized staff and services, i.e. third-level care centers, whereas there is a shortage of primary care structures providing basic headache care. This contrasts with generally accepted care policies for headaches which indicate in primary headache services is the key to providing universal headache care coverage, which is based on two arguments. The first is that the only viable way to reach the large number of people needing headache care is to implment primary care. The second is that, through appropriate levels of specific headache education, primary care can provide an effective level of primary headache care. Such a model of care requires that the different levels (i.e. primary, scondary and tertiary) are well integrated, and that the most advanced is dedicated to the minority of patients who need it [26].

It is essential to identify the necessary educational activities that will provide the practical knowledge needed to manage the most common forms of primary headaches. That is, how to treat TTH, and particularly the high frequency episodic form and the chronic form, and migraine at primary care level [68]. Referral to headache centers is for those patients with migraine, either episodic at high frequency or chronic, who do not respond to first and second-line treatments and for patients with cluster headache and other trigeminal autonomic cephalalgias, and for those with MOH secondary to CM or to chronic TTH.

The educational program should shortly cover pathogenesis, and focus extensively on clinical aspects such as symptoms and signs, differential diagnoses, and a detailed account of the available treatments, including both pharmacological and non-pharmacological ones, without forgetting lifestyle recommendations, and specifically listing those that are available and affordable in the single contexts. Educational programs must also focus on the appropriate diagnostic evaluation of primary headaches [69] and secondary headaches that can sometimes be due to potentially life threatening conditions, such as cerebrovascular conditions, neoplasms or infectious conditions [4].

Therefore, a focused training programs devoted all health professionals who wish to offer services in the field of headache should be planned with specialists from academic or third-level centers. Headache education should begin during medical training: it is a fact that the undergraduate education of medical students in headache is extremely limited and, in most universities, does not exceed 4–6 h during the medical study [70]. As a consequence, in many countries GPs cover primary health care without having any specific training on headache disorders. Headache education should be based on programs created by specialists from academic or third-level centers, also in collaboration with the scientific societies and should be offered not only to medical students and residents in neurology and other clinical disciplines, but also to all health professionals who offer services in the field of headache and who have lower-level education (i.e. up to bachelor). In this context it is worth noting that, as in many cases the first health care professional encountering a patient with headache is the community pharmacist [71] or a non-physician clinician. Therefore, pharmacists and other healthcare providers should be included in the primary health education programs in addition to GPs.

A single center clinical survey showed that, unfortunately, community pharmacist-focused educational activities did not have the expected effect [72]. However, this should not be a barrier to a large-scale training program for pharmacists specifically targeting symptomatic treatment of headaches and focusing particularly on ΜΟΗ. Indeed, pharmacists are the first ones who can intercept and educate people with frequent and recurrent headaches to limit the overuse of symptomatic headache medications and suggest medical consultation.

To sum-up, training programs for physicians who manage primary headaches and MOH have a positive influence on the way people with headache are treated [73, 74]. The duration of this postgraduate training could be condensed into repeated biannual seminars lasting 5–6 h and it might include:

- one session for pathogenesis,
- two sessions for the clinical picture and technical investigation,
- two sessions focused on the management with first line treatments, symptomatic and preventative: here, consensus recommendations covering the therapeutics of headaches should be addressed in detail.

Local universities could undertake the implementation of this program in conjunction with national headache societies or neurological societies. National pilot educational programs including the above-mentioned topics (e.g., including a one day, or a half-day seminar focused on headache in the last year of undergraduate medical training) should be carried out.

### Defining strategies for access to existing treatments for headaches in low- and middle-income countries and for facilitating the inclusion of these countries in the research and development of new medicines (RCTs or RWS) (Target 3.b)

The resources for the management of headaches in LMIC are limited, but migraine impacts the health of people regardless of geography and income [2]. Different types of barriers prevent access to health care in LMIC: healthcare-related, political, economic and/or cultural [75, 76]. Moreover, in many LMICs other major pressing health issues (such as tuberculosis, malaria, and HIV) take priority [77], despite the fact that headaches constitute the most common reason for medical encounters in LMIC [78]. The inadequate number of trained personnel, limited imaging resources and expertise in performing lumbar punctures, all contribute to decreased access to preventive treatment and to increased use of analgesics ultimately leading to MOH. The inadequate research activity on headaches in LMIC, together with limited advocacy efforts result in a dearth of information on the disability and economic loss caused by headaches to the policymakers. In the absence of an adequate support from the national health system, patients are expected to cover the cost of instrumental examinations and of preventive treatment, which is frequently prohibitive for LMIC salaries and therefore become another barrier in accessing adequate care. The belief in native medicine combined with disbelief in modern medicine – which for example often leads to believing that migraine is not a treatable disorder with a neurobiological basis [75–77] – results in failure to receive evidence-based treatment.

Recommendations for improving care and access to preventive treatment include public education, improved training of primary care physicians, potentiating the training in the residency program for neurology, and increasing the reach of neurologists to the rural areas using, for example, neuro-caravans. In addition to this, the potential offered by tele-healthcare in headache medicine, forced worldwide by COVID-19 pandemics, has to be exploited [79]. It is indispensable to increase the availability of preventive medications and decrease the use of over-the-counter analgesics: this is essential in countries in which approximately 50% of people with migraine rely on self-treatment, managing their headaches with over-the-counter drugs such as NSAIDs only [70]. The research data and prevalence studies on local populations should be augmented to gather the necessary data for formulating suitable policies.

The performance of research locally in LMIC is important to understand the cultural variations in headaches and the etiology of secondary headaches. The problems faced by the LMICs in enhancing research capacity include inadequate research personnel and research capacity, lack of integration between centers of excellence resulting in parallel research and insufficient translation of research into practice. To reduce such inequalities in research, which in turn impact on the approval of medications from local drugs administration, real-world studies generating real-world evidence should be promoted as a viable way to produce evidence on drugs’ effectiveness [80]. RCTs, which are commonly referred to as the gold standard of biomedical research, are not always the gold standard of research in different fields and produce biased results [81, 82]: real world studies have the potential to produce high-level evidence [83] and are likely to be more feasible in LMIC, although their quality is to be improved.

The strategies for improving research in LMIC include:

1. Analyzing the existing assets and improving them;
2. Collaborations and consortia between high-income countries (HIC) and LMIC to provide financial support for research and monitoring the progress;
3. Organizing conferences and webinars for training of trainers to impart technical expertise, formulating research protocols, budgeting, scientific paper writing, data management, statistics, good clinical practice;
4. Short term courses in HIC for research training;
5. Creating groups for translating research to practice;
6. Improving the access to scientific material (journals, books);
7. Increasing focus on research in undergraduate curricula;
8. Improving networking, increasing the leadership roles and increase communication between personnel involved in research;
9. Increasing researchers’ salaries to reduce brain drain to HIC and to compensate for private practice;
10. Linking departmental promotions to research experience and publications.

The barriers that impede access to preventive treatment must be suitably addressed by improving health care access and quality of care. It is equally important to develop a sustainable and systematic research capacity that shall continue to function well into the future, even after its developers have moved on. A viable way to pursue this objective lies in the systematic used of headache registries. The International Headache Society has recently released its guidelines on registries, in which core elements were presented: validated headache-specific questionnaires, patient reported outcome measures, and medical record data [84]. The use of registries will enable data sharing, which in LMIC is essential to avoid duplicating data collection effort, and the availability of reports on interventions outcome allows also to exchange data on the most efficient way to organize headache care.

### Defining strategies to develop and implement training and education in low- and middle-income countries to improve the skills of healthcare professionals for management of headaches in primary and secondary care (Target 3.c)

Management of patients living with primary headaches, or of those who present with headaches secondary to potentially life-threatening conditions, remains laden with serious deficiencies that are embedded in the lack of knowledge and skills needed to manage these clinical conditions. This includes difficulties in appropriately diagnosing and investigating such patients, and in planning adequate care pathways, which results in delayed specialist referral and continued administration of aggressive treatments that are overtly contraindicated or will be complicated by organ damage [85]. This is prevalent in LMIC where there are insufficient diagnostic tools, most importantly, neuroimaging, to confirm the causes of secondary headaches and/or identify primary headaches [86, 87]. The very few available healthcare professionals are overwhelmed with the large amount of the population requiring care, coupled with the stress of the poor socioeconomic standard of living, which is in turn aggravating TTH prevalence and severity [88].

In a bid to improve the people’s quality of life to which the presence of headaches is a major impediment [89], there has to be capacity building in skills acquisition for the management of headaches at the primary and secondary care levels in Africa and other developing countries, by scaling up and replicating training programs for strengthening health systems in the long-term. Each level of care should work within the limit of what they can offer by timely diagnosis and promptly treatment of patients, including urgent specialist referral most especially for secondary headaches which would, most times, require the tertiary level of care intervention [90]. Training programs should focus on strategies to overcome the barriers to effective service delivery by healthcare professionals, specifically [91]:

1. Knowledge and Competency Barriers, i.e. addressing the cases in which healthcare professionals do not know how to manage headaches as a result of insufficient pre-service and in-service training opportunities.
2. Structural and Contextual Barriers, i.e. addressing the cases in which healthcare professionals are not able to manage headaches, and require further training, both theoretical and practical, to improve their skills. In a situation where the workers are not provided with an enabling working environment, including tools, motivation is lost and this may lead to seeking jobs in other developed nations as is currently happening across African countries [92].
3. Attitudinal Barriers, i.e. addressing the cases in which healthcare professionals are not willing to manage patients, which in turn can have a negative impact on the effectiveness of treatment and future healthcare-seeking behaviors.

There are opportunities that can be leveraged in LMIC to develop multifaceted interventions targeting different barriers to behavioral change through active dissemination and implementation strategies. These include the younger population and lower cost of living, which are of advantage in labor force and innovation [93]. This vibrant group of the population should be judiciously recruited for training in the context of the available resources by adopting the “Task Shifting and Task Sharing” policy to address the progressive shortage of personnel in the healthcare sector [94]. Therefore, for the successful achievement of SDG 3, in relation to headache management, this framework of five elements for implementing transferable and sustainable training programs for healthcare professionals in LMIC is recommended [95, 96]:

1. Implement the local headache school by means of a short course format which can consist of 3 sessions, 1–2 weeks per session. This will minimize interference with the healthcare professionals’ duties while allowing for focused didactic training and group work sessions on practical management components and specific areas of need. These include the training on the use of primary headache clinical diagnostic criteria, but also system-level actions;
2. Implement or create where lacking the local headache group by means of on-site (hospital) training and mentoring, through learning by doing approaches rather than through classroom lectures alone, which will allow participants to practice the reading and interpretation of the diagnostic tools in their own work setting, with the participation of colleagues and the support of on-site mentors;
3. Integrate the “training of the trainers” methodology by collaborating with local academic institutions that are willing and able to upgrade and maintain the training by integrating it into their curricula. There should be continuing medical education which incorporates classroom presentations, lectures, conferences, and educational materials including audit/performance assessment with feedback outreach visits;
4. Secure support from the Ministries of Health to ensure participation in the training by using the Theory of Change approach;
5. Train rural healthcare workers living in the rural setting by applying the principles of talents management strategies [97] to improve recruitment and retention of skilled healthcare providers in rural underserved areas with consequent improvement in access to healthcare at the primary care level. This is achievable by the implementation of the Rural Medical Education Program [98].

### Defining strategies for an inclusive and global alliance against headache disorders among headache healthcare professional working parties to respond to public health unmet needs in headache area (Target 3.d)

The ASD-2030 is calling for a whole-of-society approach to respond to development challenges that are increasingly pressing, complex and interrelated. Such a call requires a response that is based on a joint committment of different stakeholders aimed to reach long-term solutions for populations which will not leave anyone behind. In the field of headache disorders the call to attain SDG 3 must include the creation and implementation of a global partnership of relevant stakeholders. The relation of SDG 3 with neurological disorders, in particular headaches, and the way in which its achievement intersects with the future of neurological practice have not been comprehensively examined to date, and highlighting some of the key elements to define a global strategy also allows headaches to be a case model for other diseases [99].

The approach of the 2030 Agenda for Sustainable Development can be applied to many fields of health and among them, neurology is leading the transformation thanks to several actions. The strategies that need to be implemented are not exclusively health-related: rather they will have to embrace an holistic approach to global health, which recognises the different components of populations' health and well-being. Access to health services need to address promotion, protection and recovery - whenever possible - of neruolgical and brain health. To pursue this aim, strategies will likely need to be designed and implemented by different stakeholders, including healthcare specialists and end-users [100]. There are several pillars through which SDG 3 could be achieved by the headache community. Campaigns such as “Lifting the Burden” (LTB), the Global Campaign Against Headache attracted attention and developed policies to reduce the burden of headache disorders. LTB, a collaboration between the World Headache Alliance, the International Headache Society, the European Headache Federation and the World Health Organization (WHO), aimed to raise awareness of headache burden and implement health-care solutions to reduce these burdens worldwide.

In May 2022, during the 75th World Health Assembly in Geneva, WHO Member States unanimously approved the Global Action Plan on Epilepsy and Other Neurological Disorders (WHO-GAP-ND) [101], which delineates aims, objectives and targets that can be used by the headache global community to define precise targets. To meet the global targets, the WHO-GAP-ND includes a set of actions for the WHO Secretariat to be carried out, as well as for Member States, in a set of areas, including health promotion, prevention, care, treatment and rehabilitation as well as education and research. It is intuitive, considering headaches’ prevalence, that good health and well-being at the population level must include reduction of headache burden in terms of freedom from, mitigation, and treatment of headache disorders to the highest degree possible. SDG 3 put emphasis on recognizing the environmental risks for health that can be addressed accounting an “health for all” perspective in all policies, including care for disorders, prevention and health promotion. To get to this point, the health sector needs to be stregthened, mechanisms of governance need will need a profound revision in the way they organize healthcare, and communication between different stakeholders will need to be improved [102].

Achieving the ASD-2030 and its 17 SDGs will require the collective effort. New partners and more ecnomic resources will be needed, together with an enhanced approach to available resources' use: in this way, the impact can be maximised. Stakeholders will need to pursue development objectives unders a strong co-operation perspective, so that future joint actions can be succesful. This is why all headache scientific and patient advocacy organizations should join forces to develop a common plan of global action. To properly address headaches' burden, a joint actions from a variety of stakeholders - including patients, clinicians, policy makers, and the general poplation - is needed [100]. Such a global joint action has to be aligned and well coordinated across countries in order to respond to national development priorities and that development planning.

Results at country level should focus on priority areas, so that success and drawbacks are defined by the results of actions, and not by the kind of actions carried out: in this way, decisions on further implementation plans can be based upon the discussion on the challenges faced throughout the implementation of the actions [103]. Concerning increasing sustainability, two crucial elements for development strategies of SDGs are inclusiveness and results orientation. Actions in single countries are ruled by the governments that are responsible for implementation in each country. Information on the effectiveness of actions should however be made available to other contries and governments. In this way, the different stakeholders - including civil society, private sector and politicians - that are co-responsible for implementing country-level actions, can rely on such sharing of good practices. In turn, this leads to better development plans, at the sime time providing room enough to engage new actors that might enrich the strategies in course of definitions with origianl ideas and inputs [104]. The involvement of stakeholders, in order to be effective, in fact requires the ability to move beyond simple consultation to embrace an inclusive participation approach. Such an approach requires that opportunities to participate in open discussions, sharing information about opportunities for engaing in different actions, and addressing issues that might negatively impact on inclusive participation.

The global incidence, prevalence and burden of headaches, the definition of sustainable health care pathways, the outcomes of people living with headaches, the training of future health care professionals with expertise in the management of headache, can be interlinked, directly or indirectly, with programming for the SDGs and their eventual achievement. Working together for the development of “headache friendly health policy” means working on agreed objectives and jointly set milestones, to achieve predetermined targets and results.

Overarching strategies would start by defining global common goals to control and eventually reduce the number of people living with headache disorders, their impact on personal lives and on societies trying to develop and share sustainable data systems. It will be essential for all stakeholders to learn to promote cross-country learning exchanges which should address the following objectives:

1. Opening a discussion with Scientific Journal editors and publishers on the prices of Open Access, so as to avoid exclusion of LMIC from publications;
2. Facilitating access to funding for researchers working in LMIC;
3. Increasing the use of e-education with shared curricula to increase inclusiveness of all possible health professionals in all countries, including translation of management guidelines in several languages;
4. Promoting the presence of balanced and representative gender, age, and ethnicity equity in all the meetings, congresses, as well a balance between young and more senior researcher as well as speakers from HIC and LMIC, and patients’ representatives able to co-create opportunities for joint public health actions;
5. Increasing the use of telemedicine, virtual learning, virtual congresses to increase global participation;
6. Studying common solutions to allow innovative drugs to be available also in LMICs.

All these actions if implemented will make the headache field more sustainable and will allow the headache community to work towards the SDG 3 achievement in 2030.

## Conclusions

This paper aimed to provide an analysis and a proposal of political actions to be taken to achieve the main goal of SDG 3, namely ensuring healthy lives and promote well-being for all, at all ages, specifically focusing on what could and should be done in the field of headache disorders. Headache disorders are among the most prevalent, particularly migraine is prevalent among women aged below 50, and disabling conditions both in HIC and LMIC, and constitute one of the most common reasons for a medical encounter. Thus, addressing the burden of headache disorders has the direct consequence of improving the health of populations, as well as of improving the productivity of people aged below 50, women in particular.

Our analysis pointed out several elements, including moving forward from frequency-based parameters to define headache severity, recognizing and treating the overuse of medications early, promoting education of healthcare professionals with specific training on headache before and after graduation, and promoting a culture favoring the recognition of headaches as disease with a neurobiological basis where this is not recognized. Such elements are, in a sense, prerequisites for the organization of healthcare systems at different levels of care, from primary to third-level one. This is of particular importance, as the majority of patients with headache disorders can be appropriately treated at primary (or even pharmacy) level, thus leaving higher-levels of specialized care to those who need them. What is clearly to be pointed out here is that our analysis is aimed to set out the stage for policy development and not to present direct and concrete actions. Action need in fact to be tailored on the specificity of different countries and on the economic features of the contexts in which actions have to be implemented. Actions need to be realistic in order to be achievable and guidelines for action need to be based on agreed goals: thus, practical solutions will necessarily come at a later stage.

A joint effort is needed to pursue such objectives, and the actors that need to be involved include policy makers, academics with specific expertise in headache disorders, and representatives of patients and of scientific societies. These objectives are surely challenging: however, achieving them is not only feasible, but necessary to reduce the burden of disease at the global level.

Making headache care more sustainable will improve brain health, and efforts to optimize brain health require multi-stakeholder collaborations that should be integrated across all sectors of society, spanning from healthcare to education and including, for example, emplyment and governance. Robust investment will be needed, but a return of investment is envisaged by the succesful actions towards implementation of brain health across all ages, which in turn will lead to better health outcomens and well-being, as well as reduced population-level disability and burden [105]. Multisector engagement and collaboration are urgently needed to move the brain health agenda forward for all people. Reducing the burden of headache will contribute to increase global brain health and to reach SDG 3.

## Data Availability

Not applicable.
